# Soft, Comfortable Polymer Dry Electrodes for High Quality ECG and EEG Recording

**DOI:** 10.3390/s141223758

**Published:** 2014-12-10

**Authors:** Yun-Hsuan Chen, Maaike Op de Beeck, Luc Vanderheyden, Evelien Carrette, Vojkan Mihajlović, Kris Vanstreels, Bernard Grundlehner, Stefanie Gadeyne, Paul Boon, Chris Van Hoof

**Affiliations:** 1 KU Leuven-University of Leuven, Departement of Electrical Engineering, Kasteelpark Arenberg 10, Leuven 3001, Belgium; E-Mail: Chris.VanHoof@imec.be; 2 IMEC, Kapeldreef 75, Heverlee 3001, Belgium; E-Mails: Maaike.OpdeBeeck@imec.be (M.O.B.); Kris.Vanstreels@imec.be (K.V.); 3 Datwyler Sealing Solutions, Industrieterrein Kolmen 1519, Alken 3570, Belgium; E-Mail: luc.vanderheyden@datwyler.com; 4 Department of Neurology, Ghent University Hospital, 1K12IA De Pintelaan 185, Gent 9000, Belgium; E-Mails: evelien.carrette@UGent.be (E.C.); Stefanie.Gadeyne@UGent.be (S.G.); paul.boon@uzgent.be (P.B.); 5 Holst Centre/imec-nl, High Tech Campus 31, 5656 AE Eindhoven, The Netherlands; E-Mails: Vojkan.Mihajlovic@imec-nl.nl (V.M.); Bernard.Grundlehner@imec-nl.nl (B.G.)

**Keywords:** flexible polymer dry electrode, material optimization, impedance, high quality biopotential recordings, ECG, EEG, high user comfort, conductive polymer

## Abstract

Conventional gel electrodes are widely used for biopotential measurements, despite important drawbacks such as skin irritation, long set-up time and uncomfortable removal. Recently introduced dry electrodes with rigid metal pins overcome most of these problems; however, their rigidity causes discomfort and pain. This paper presents dry electrodes offering high user comfort, since they are fabricated from EPDM rubber containing various additives for optimum conductivity, flexibility and ease of fabrication. The electrode impedance is measured on phantoms and human skin. After optimization of the polymer composition, the skin-electrode impedance is only ∼10 times larger than that of gel electrodes. Therefore, these electrodes are directly capable of recording strong biopotential signals such as ECG while for low-amplitude signals such as EEG, the electrodes need to be coupled with an active circuit. EEG recordings using active polymer electrodes connected to a clinical EEG system show very promising results: alpha waves can be clearly observed when subjects close their eyes, and correlation and coherence analyses reveal high similarity between dry and gel electrode signals. Moreover, all subjects reported that our polymer electrodes did not cause discomfort. Hence, the polymer-based dry electrodes are promising alternatives to either rigid dry electrodes or conventional gel electrodes.

## 1. Introduction

Biopotential signals are the result of the electrochemical activity of certain cells of the nervous, muscular or glandular tissue. When one cell is triggered, ion exchange occurs through the membrane of this cell, creating a so-called action potential. A biopotential signal is the action potential from one cell or the average electrical activity of a group of cells, and such signal can be monitored on various locations of the human body [1–3]. The average activity of the brain cells can be monitored on the scalp, and this signal is called electroencephalography (EEG). The electric activity of heart cells is monitored by electrocardiography (ECG).

Monitoring biopotential signals, such as ECG and EEG, provides important information about certain health-related conditions of a person, which opens a broad range of applications [4,5]. Wearable devices monitor ECG to trace heart rate during exercising. During clinical ECG monitoring, the biopotential signals are studied in greater detail, in order to diagnose cardiovascular diseases. EEG monitoring on patients suffering from epilepsy is applied to register seizures, essential to diagnose workup and for seizure classification [6]. EEG monitoring offers also interesting possibilities for brain computer interface (BCI) applications [7]. Motion disabled patients are able to control their wheelchairs, and communication disabled patients can spell words by BCI techniques based on EEG signal recording [8].

In this paper, the electrodes used for the ECG/EEG measurements are subject of investigation. An important criterion for suitable electrodes is low impedance to limit the noise generated during signal recording, jeopardizing the accuracy of the obtained biopotential signals. The stratum corneum (SC), which is the top layer of the skin has a high impedance [1]. Therefore, wet gel electrodes are very interesting: the gel hydrates the SC, hence creating an ionic path between the metal part of the electrode and the skin below the SC layer, which makes the transduction of ionic current into electric current easier. Therefore, the gel lowers the skin-electrode impedance. Furthermore, the flexible gel stays in good contact with the skin during movement, hence the impedance also remains low when the subject is moving. Therefore, gel electrodes provide high quality signal recording with limited motion artifact disturbances, which makes them very popular for biopotential recording. However, various drawbacks of the gel are also well known [9,10]. Before biopotential monitoring, skin preparation is often needed to improve signal quality: abrasive gel and cleaning alcohol are used to remove some part of the SC layer. For long term EEG recoding, electrodes have to be glued on a well-defined position on the scalp. Then the electrodes are filled with conductive gel. This time-consuming EEG setup has to be done precisely by an expert. During the EEG monitoring, signal degradation will occur due to gel drying out, and skin irritation is often observed. After EEG recording, electrode removal is difficult and time consuming. If electrodes are glued on the scalp, the removal is often even painful. For short period monitoring, electrodes can be clamped on the scalp by a cap, requiring shorter setup and removal times, but being uncomfortable to wear.

**Figure 1.**
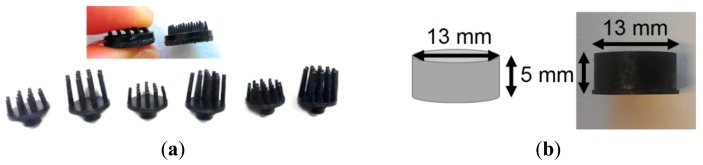
(**a**) Pin-shaped and (**b**) cylinder-shaped conductive polymer dry electrodes.

To avoid these drawbacks of wet electrodes, various types of dry electrodes have been introduced, as discussed in various publications [11–22]. Contact and non-contact electrodes are the two main categories of dry electrodes [13]. Non-contact electrodes are capacitively coupled to the skin, hence, these electrodes give a very small signal amplitude and are highly sensitive to motion artifacts since motion will change the skin-electrode capacity [14]. For the direct contact electrodes, two types exist: electrodes with microscale needles on the surface which just penetrate the SC, and electrodes which are in direct contact with the skin but without penetration. Since microscale needles are conductive (conductive bulk material or coating), they will lower the overall electrode-skin impedance. In order to function well on hairy skin, contact electrodes are often equipped with macroscale pins (e.g., Figure 1), since the hair can be positioned in the space in between the pins and hence an improved electrode-skin contact results. Various authors have reported their contact dry electrode work. Regarding penetrating contact electrodes, nanoscale needles made of carbon nanotubes are reported in [15], while microscale tips have been fabricated using a silicon wafer with conductive coating [16,17]. Macroscale pins for non-penetrating electrodes for hairy skin were developed using non-conductive materials with conductive coating [18]. For non-hairy locations, polymer foam electrodes with metal coating or conductive textile electrodes can be used [19,20]. Besides, in order to enhance the electrode-skin contact, a tattoo-like epidermal sensor-system was developed for biopotential signal recordings [21]. This tattoo-like system consists of dry electrodes in the shape of a stretchable metal mesh on thin silicone which is attached very well to the skin. Due to the stretchability of this ultrathin sensor, very good electrode-skin contact is obtained on non-hairy skin. So called “semi-dry” electrodes were also developed; such electrodes release an electrolyte for skin hydration during use [22].

Nano- and microscale electrodes for SC penetration are expensive due to their fabrication process. Moreover, since these electrodes penetrate into the SC, they might cause irritation and infections, especially in case of breached skin [10]. Moreover, additional requirements regarding material biocompatibility have to be respected since the needles will easily break during use. Therefore, contact electrodes for SC penetration are not popular for use in commercially available EEG systems.

Regarding pin shaped dry electrodes, full metal electrodes have the lowest impedance but due to their rigidity combined with the pressure essential for good skin contact, they are painful during use [23]. Softer non-conductive polymer electrodes coated with a metal layer are more comfortable, but the coating easily flakes off, resulting in high resistivity of the electrode.

In this paper, dry electrodes fabricated from flexible conductive polymer get all attention. Such electrodes overcome the drawbacks of wet gel electrodes and of the various types of dry electrodes mentioned above. The elastic properties of the conductive polymer ensure user comfort, while the conductivity of the polymer avoids any layers from flaking off during use. Electrodes with various pin shapes (length, width, pin density) are fabricated for different applications. Various additives were added into the polymer, and additive type and quantity were optimized to obtain low contact impedance and good mechanical properties for better comfort and skin contact. Impedance measurements on phantoms and human skin were carried out for impedance characterization. Nano-indentation tests were used for hardness and elastic modulus characterization of these electrodes. Moreover, compression tests were carried out to investigate the flexibility of pin-shaped electrodes. Finally, the optimized conductive polymer electrodes were used for ECG and EEG recording, and the obtained biopotential signals were compared to those obtained using conventional wet electrodes, by studying correlation, coherence and signal to noise (SNR) ratios.

## 2. Electrode Fabrication

### 2.1. Shape Designs

The shape of electrode was designed using Solid Edge (Siemens PLM Software, Plano, TX, USA). For the top side of the electrode, being the side in contact with the skin, both flat and pin-shaped were designed. For the bottom side of the electrodes various shapes were designed to integrate the electrodes with various types of ECG/EEG recording systems. The designs were used to make the electrode molds. The various material mixtures described in next subsection underwent compression molding, forming the electrodes as shown in Figure 1. The base planes of these electrodes have diameters from 10 to 13 mm and pin lengths from 2 to 8 mm. Cylindrical shaped electrodes with 13 mm diameter and 5 mm height were also fabricated for each material mixture, in order to test their mechanical properties and other material characterization tests, these electrodes are further called “bulk electrodes”. The type of electrode used for each particular test will be mentioned in each section of this text.

### 2.2. Materials

In order to obtain the optimum properties regarding material conductivity, hardness, flexibility and ease of fabrication, various additives were mixed in an ethylene propylene diene monomer (EPDM) matrix and the resulting polymer was tested as electrode material. Several types of conductive additives are tested, such as carbon, stainless steel fibers and carbon nanotubes. The polymer material containing ∼45% of carbon was tested for cytotoxicity. For this polymer dry electrode, cytotoxicity is determined by testing the polymer material after molding, since the molding process might influence the chemical/physical properties of the material. Two cytotoxicity tests were performed: (1) *in vitro* cytotoxicity tests according to ISO 10993-5—(Part 5: Tests for *in vitro* cytotoxicity) and (2) USP-based tests (36-NF31:2013<87>, Biological Reactivity Test, *In Vitro*). Both test results show this material is non-cytotoxic. Tests are performed by Toxikon Europe (Leuven, Belgium), an accredited analytical test lab assisting in medical device development, product safety and regulatory compliance [24]. After all optimizations, once the final composition of the polymer material is fixed, more tests will be performed, such as skin sensitization and intracutaneous reactivity.

## 3. Experimental Section

### 3.1. Optimization of Conductivity and Mechanical Properties of Polymer Dry Electrodes

As explained before, conductive additives were added into the EPDM matrix to make the material conductive. The type and amount of additives influence not only the conductivity, but also the mechanical properties of the electrodes. To determine an optimized material composition, impedance measurements and nano-indentation tests were carried on electrodes fabricated from various polymer compositions.

#### 3.1.1. Impedance Measurements of Various Polymer Compositions of Bulk Electrodes for Conductivity Optimization

First, the cylindrical shaped bulk electrodes (Figure 1b) with various carbon contents in the polymer are used to study the material conductivity. Two Au-coated plates were used to contact the top and bottom surface of each sample. A clamp was used to stabilize the bulk sample in between the two metal plates. An IVIUM potentiostat (Ivium Technologies B.V., Eindhoven, The Netherlands) and integrated impedance analyzer were used for the impedance measurements. Four electrodes (reference, counter, sense and working electrode) can be connected to the potentiostat in order to use a 4-electrode setup. For the material conductivity tests, a 2-electrode setup was selected for the measurements. Therefore, the reference and counter electrodes of IVIUM were shorted and connected with the first metal plate while the sense and working electrodes of IVIUM were shorted and connected with the other metal plate. The impedance of conventional ECG wet electrodes (ARBO H124SG, Kendall, Mansfield, MA, USA) was measured for comparison. To do this, two wet gel electrodes were placed back to back (both gel parts sticking together) and the electrode snaps were used as connectors for the electrode input of the IVIUM tool.

A 25 mV signal was generated by the IVIUM potentiostat and impedances were measured when the signal swept through frequencies from 0.1 Hz up to 10 kHz. In this paper, only the impedance at 10 Hz is shown in the graphs to reduce the obtained information down to the frequency band of interest for biopotential signals, which is a few to hundred Hz [3]. For correct comparison of various electrodes, the size of the electrode should be taken into account. Obviously, a larger contact area between the electrode and the skin will result in a lower contact resistance, but a large contact area is not practical and limits the resolution of the recording. Hence, the measured electrode impedance is always normalized towards the skin area covered by the electrode. As such, the normalized impedance is independent of the electrode geometry but is an intrinsic property of skin-electrode contact for the electrode under investigation [25].

#### 3.1.2. Impedance Measurements of Various Polymer Compositions of Pin-Shaped Electrodes on Phantoms and Human Skin for Conductivity Optimization

When electrodes are used for biopotential measurements, the impedance of the system consists of the impedance of the combination of an electrode with the skin. Hence, the skin-electrode impedance is of utmost importance. Since this value will differ from subject to subject and even within time for the same subject, the combination of the electrode with (stable) phantom surfaces is also evaluated, as a reference. A platinum metal film and a cloth wetted with electrolyte solution were used as phantoms to utilize well-controlled and stable conditions for impedance characterization. Hence, electrodes with various carbon contents were placed on phantoms and on the skin of test subjects for impedance characterization. The measurements were also carried out on human skin of different subjects to investigate the skin-electrode impedance variation between subjects.

A 3-electrodes set-up of the IVIUM potentiostat was used to acquire the impedance of skin/phantom-electrode combination [26]. Conventional wet electrodes were used as counter and reference electrodes for the IVIUM tool. They were placed 10 cm apart from each other on the skin/phantoms, and left in place for 30 min before starting the measurements, in order to allow impedance stabilization at the contact interface. The electrode under test was connected with the working and sense cables of IVIUM and placed next to the reference electrode. As working electrodes, the polymer electrodes were used, but a wet gel electrode was also characterized in order to compare this result to the various polymer electrode results.

**Figure 2.**
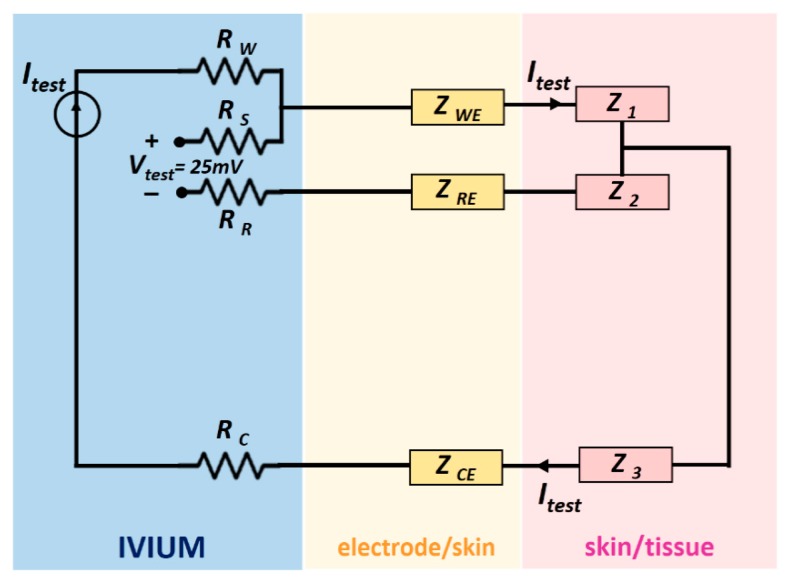
Schematic of the three-electrode set up for impedance measurement by the IVIUM potentiostat.

During the measurements, the potentiostat controlled a 25 mV AC signal between the working and reference electrodes, the signal frequency varied from 0.1 Hz up to 10 kHz. Since the internal impedance of the voltage meter of the IVIUM is over 1000 GΩ, the current passed mainly through the working and counter electrodes and the skin and tissue between them. The related impedances are labelled as Z_WE_, Z_1_, Z_3_ and Z_CE_ in the schematic shown in Figure 2, where Z_1_, Z_2_ and Z_3_ stands for the impedance of the material (phantoms or tissue) in between electrodes respectively.

The impedance Z_WE_, being the impedance of the skin/phantom-working electrode, was computed internally by the IVIUM. As explained in the last subsection, only the impedance values corresponding to a 10 Hz input signal will be shown in this paper. For correct comparison of various electrodes, the impedances are always normalized to the skin area covered by the electrode. The skin area of a conventional wet electrode is the gel area, while that of pin shaped electrode is the total base area.

#### 3.1.3. Mechanical Properties of Various Polymer Compositions

The hardness and elastic modulus of materials were identified by nano-indentation tests while the flexibility, which is a combination of electrode materials and designs, was analyzed by the load-displacement curves of compression tests [27].

Nano-indentation tests of the cylinder-shaped polymer electrodes (Figure 1b) with various carbon contents were carried out using a nano-indenter XP system (MTS Systems Corporation, Eden Prairie, MN, USA), equipped with a standard three-sided pyramid diamond indenter tip (Berkovich tip). Hardness was determined using the loading cycle of the indentation test, while the elastic modulus was determined from the unloading cycle. From the experimentally obtained load-displacement curves, the elastic modulus (*E*) and hardness (*H*) of these electrodes were calculated based on their relationship to the contact area (*A*) and the measured contact stiffness (*S*) as indicated in Equations (1) and (2):

(1) $$H=\frac{P_{\max}}{A}$$

(2) $$S=\beta \frac{2}{\sqrt{\pi }}\sqrt{A}{\left[\frac{1-\nu^{2}}{E}+\frac{1-\nu_{i}^{2}}{E_{i}}\right]}^{-1}$$

where *P_max_* is the maximum applied force, *A* is the projected contact area of the indenter with the sample surface, β = 1.034, ν is the Poisson's ratio of the sample and *E_i_* (1140 GPa) and ν*_i_* (0.07) are the elastic constants of the diamond indenter tip. Finally, the contact area (*A*) was determined from the indenter tip shape calibration, as described in [28,29].

Compression tests were carried out to investigate the flexibility of using pin-shaped electrodes containing various amount of carbon content (28.2%, 37% and 43.6%). These tests were performed using a high precision micro-mechanical test system with full computer control and data analysis. It included an ultra-high-resolution linear actuator providing linear motion up to 50 mm with a resolution 20 nm. For the compression tests, the maximum applied load was 15 N to avoid severe sliding of the pins. The displacement rate was fixed to 25 μm/s for all compression tests [27].

### 3.2. ECG Recording

#### 3.2.1. Electrodes Set-Up and Recording System

**Figure 3.**
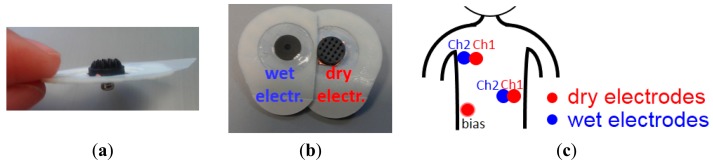
(**a**) Conductive polymer dry electrode connects with gel removed wet electrode; (**b**) A pair of wet and dry electrode; (**c**) Electrode locations for ECG measurements.

The optimum conductive polymer electrodes containing ∼50% of carbon and having 2 mm pin length and 10 mm base size were used for ECG recordings. To connect the polymer dry electrode to the recording system and to place the dry electrode on chest, a conventional wet gel ECG electrode (Meditrace 200 Series ECG Electrodes, Kendall) was taken, and the gel was removed leaving only the snap and the skin adhesive part as attachment for the dry electrode. The backside of the dry electrode was attached to the snap using copper double sided tape as shown in Figure 3a. In order to correctly compare the signal of a dry electrode with that of a wet electrode, two conventional wet electrodes were placed next to two dry electrodes to obtain two very similar ECG signal (see Figure 3b). Two such pairs of wet-dry electrodes were placed on the subject's chest and combined with one dry electrode as bias signal at the chest locations shown in Figure 3c. ECG are signals in the mV range, hence these signals are strong enough to be acquired directly without pre-amplifier between the dry electrodes and the ECG recording system. An analog front-end ECG recording board designed in imec was used as the recording system [30]. This recording board equipped with programmable 4th order low pass filter (LPF). A 200 Hz LPF was selected for the recordings in this paper. The subject was sitting on a chair and was not moving during the recordings. Both wet and dry signals were recorded simultaneously.

#### 3.2.2. Signal Analysis

After signal acquisition, 50 Hz power line interference and breathing muscle artefacts can be present in the obtained wet and dry ECG signals, hence both signals were filtered by a 50 Hz notch filter with a bandwidth of 0.25 Hz and then by a first order Butterworth high pass filter with a frequency of 0.5 Hz [31], in order to remove both signal disturbances. The two signal filters were designed in Matlab. To compare the signals, the correlation of filtered wet and dry signals is computed in Matlab by a cross-covariance function.

### 3.3. EEG Recording

The conductive polymer electrodes containing ∼44% of carbon and having 5 mm pin length and 13 mm base diameter, were used for EEG recordings. The electrodes containing ∼50% carbon were not used for the EEG monitoring due to their hardness and limited elasticity, which may cause discomfort for the test subject.

#### 3.3.1. Recording System and Active Electrodes

The EEG measurements were performed in an EEG recording room at Ghent University Hospital (UZ Gent) by using Brain Quick SD LTM 64 BS (micromed S.p.A, Mogliano Veneto, Italy) as recording system.

The amplitude of EEG signals is in the μV range, so an active circuit next to the polymer dry electrode is needed to compensate for the high impedance of our electrode [32,33]. Such a so-called “active electrode setup” helps reduce the interference from the environment, such as 50 Hz noise and signal disturbance caused by cable movement. Amplification of the active circuit can be considered to achieve higher signal to noise ratio (SNR), but in this paper, an active circuit without amplification (acting as a unity gain buffer amplifier) was used for the EEG recordings. This unity gain buffer helps to convert the high output impedance of the dry electrode to low output impedance, which can also reduce the interference from the environment.

**Figure 4.**
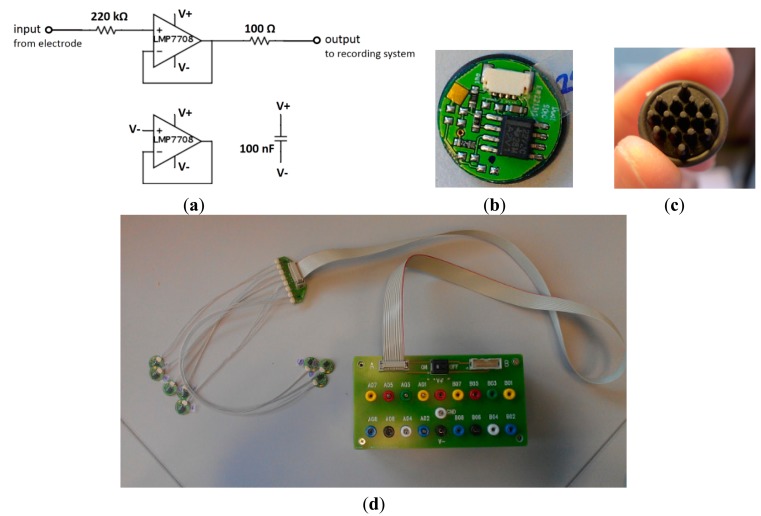
(**a**) Schematic of active circuit; (**b**) PCB of the active circuit; (**c**) Polymer electrode attached to the backside of the PCB; (**d**) The terminal board which can accept up to 16 channels of active electrodes.

Our active circuit was modified based on one of the active electrode circuits from OpenEEG website [34]. The schematic of the active circuit is shown in Figure 4a. The circuit contains an op-amp, a body safety resistor, a decoupling resistor and a capacitor next to the op-amp to stabilize the power supply. The backside of the PCBs shown in Figure 4b was coated with Au, where the conductive polymer electrodes could be connected as shown in Figure 4c. Up to 16 pieces of active circuit could be connected to a terminal box, which provides the 9 V power supply and connects the signal of each electrode to the Brain Quick EEG recording system, see Figure 4d.

#### 3.3.2. Electrodes Locations and EEG Recording

**Figure 5.**
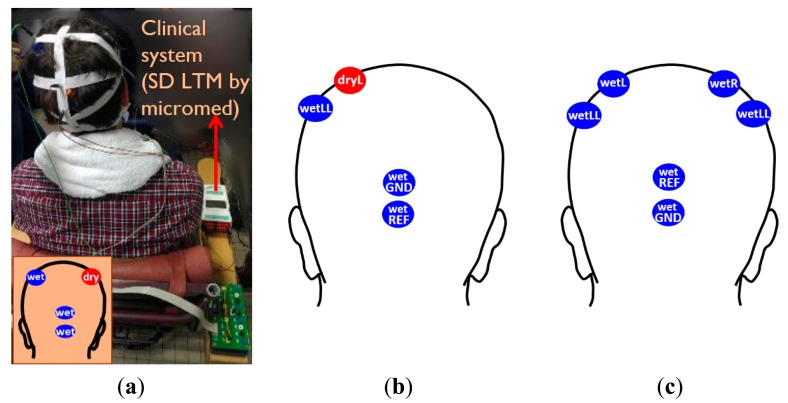
(**a**) Wet and dry electrodes were mounted on the scalp by elastic bands; (**b**) Electrode location of dry electrode recording; (**c**) Electrode location of reference recording.

The assembled polymer dry electrodes were mounted on the subject's scalp by using elastic bands as shown in Figure 5a. The length of each elastic band is adjustable, in order to realize a suitable fit for most common adult head sizes. We adapted the length of elastic bands according to each subjects' head sizes when it was in use. In order to compare the signals between wet and dry electrodes, a conventional wet bridge electrode (wetLL) was placed at ∼3 cm distance from the dry electrode (dryL), taking care to avoid any gel smearing. Two other wet bridge electrodes were used as reference (wetREF) and ground (wetGND) electrodes and were mounted on the occipital area of subjects as shown in Figure 5b. This location was chosen to avoid expected signal disturbance from eye blinking when placing the reference and ground electrode closer to the eyes.

During the recording, the subjects were asked to sit on a chair and relax with their eyes open or closed. “Eyes open” and “Eyes closed” sessions lasted 10 s respectively and were repeated three times during the measurement. The registered signal of each “eyes open” and “eyes closed” segment from one subject was analyzed.

There is always discussion about how to compare two EEG signals. Two approaches are possible: two electrodes located in close proximity measured simultaneously, or two electrodes located at the same position but measured consequentially. For both approaches, the signals are not expected to be exactly equal [11]. In this work, we determined also the highest expected correlation of two standard wet bridge electrodes placed in close proximity, during a so-called a reference recording test, in order to compare this reference recording result with the obtained correlation between signals acquired by a wet bridge electrode and a polymer dry electrode placed in close proximity. The dry electrode tests described above were performed before the reference recording test, in order not to hydrate the skin prior to the dry electrode test. All wet and dry electrode locations used to perform the dry electrode test and the reference recording test are indicated in Figures 5b and c, respectively. A high correlation of the wet electrodes signals from the reference recoding can be expected, due to short distance between the highly conductive wet gel electrodes.

#### 3.3.3. Signal Analysis

The data was filtered backward and forward by a 2–30 Hz Chebyshev type II bandpass filter to eliminate expected distortion [35]. First, correlation between signals obtained by wet and dry electrodes was calculated. Second, coherence calculation was also carried out for wet and dry electrodes by using *mscohere* function in Matlab for signals between 8 and 13 Hz. This frequency range was chosen since typically easily recognizable “alpha waves”, having a frequency between 8 and 13 Hz, are produced in the brain of most persons when eyes are closed. Last, the signal to noise ratio of the “eyes-closed signals” was calculated by the ratio of power spectrum density of alpha waves signal to that of the signal in 2–30 Hz, as described in [36].

### 3.4. User Comfort and Skin Irritation

During the impedance evaluation and ECG/EEG measurements, the dry electrodes contacted the skin for at least half an hour, corresponding to short-term use of the electrodes. The subjects were asked to evaluate user comfort after the measurements, and the skin contact area was observed for irritation or erythema.

Two long-term impedance experiments focusing on user comfort and skin irritation were performed lasting up to several days. In the first experiment, a coin-shaped electrode (no pins) was mounted on the upper arm of a subject for six full days, using a sticker (similar as the sticker used for conventional wet gel ECG electrodes), as indicated in Figure 3a. The skin condition was observed after the 6-day experiment. The second long term experiment was performed on the same subject but using an electrode with pins of 5 mm length for 60 h. An elastic bandage is used to fix the electrode on the upper arm, as a replacement for the sticker used during the first long term experiment. The electrode was removed daily for maximum one hour to allow showering. Afterwards, it was placed back at exactly the same location. The skin condition was observed daily during the experiment as well as when the experiment was finished.

## 4. Results and Discussions

### 4.1. Optimization of Conductivity and Mechanical Properties of Polymer Dry Electrodes

#### 4.1.1. Impedance Measurements of Various Polymer Compositions of Bulk Electrodes

**Figure 6.**
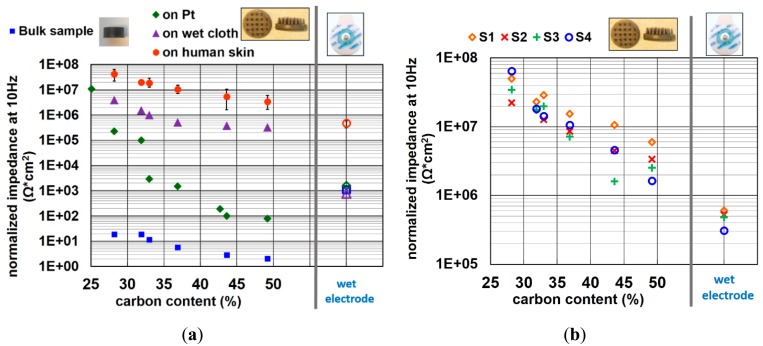
Normalized impedance at 10 Hz signal frequency of conductive polymer electrodes with various carbon content (**a**) of materials themselves, on phantoms and on human skin; (**b**) on forearm skin of four different subjects (S1–S4).

The normalized impedance of bulk electrodes at a signal frequency of 10 Hz is depicted as blue data points in Figure 6a. As expected, the impedance decreases with the increasing of carbon content.

#### 4.1.2. Impedance Measurements of Various Polymer Compositions of Pin-Shaped Electrodes on Phantoms and Human Skin

In Figure 6a, the impedance of electrodes containing various amounts of carbon on platinum (Pt), on a cloth wetted by electrolyte (wet cloth), and on the forearm of four different subjects are shown. The data of impedance on human skin shown in this figure is the averaged value of four different subjects. The error bars reveal the variation between the different subjects. It is obvious that the trend of decreasing impedance with increasing carbon content is similar for all substrates. When comparing the measurement on human skin with those of bulk electrodes, the impedance is increased by the order of 10^6^ Ω for all rubber material compositions.

It is reported that the impedance of skin, mainly contributed by the SC layer, is around a few hundred KΩ to 1 MΩ per square centimeter at 10 Hz input signal frequency [37–39]. This explains why the impedance of dry electrodes on human skin increases approximately by the order of 10^6^ Ω. For wet electrodes, the impedance variation between measurements on human skin with those of back-to-back assembly is rather small because of the skin hydration effect of the gel, which reduces the impedance of SC layer. The impedances of the wet electrode obtained by back-to-back measurements, on Pt and on wet cloth phantoms have all similar values, since this impedance is generated by the gel electrode itself, instead of the interface of the gel to the measurement surface. The trend of impedance on Pt is not as well correlated as it is on other surface. This might be because the Pt metal film has a rather flat and rigid surface. Though the force of placing the electrodes on Pt is well controlled, it is hard to estimate the bending direction and amount of contact area when the pins contact the Pt surface. A small non-uniform force can cause an important change of contact area. This might explain the nonlinear decreasing of impedance when increasing the carbon content on Pt.

The impedance of each subject can be seen in Figure 6b. Although the impedance variation between all subjects is considerable, the trend of decreasing impedance with increasing carbon content is strongly pronounced for all subjects. The impedance of the electrode with highest carbon content is ∼10-fold higher than that of conventional wet electrodes on human skin.

#### 4.1.3. Mechanical Properties of Various Polymer Compositions

**Figure 7.**
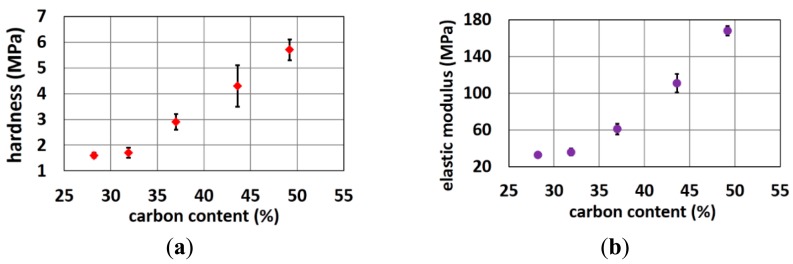
(**a**) Hardness and (**b**) elastic modulus of polymer cylinders with various carbon content.

Figure 7 shows that the hardness and elastic modulus increases when carbon content increases. These properties limit the amount of carbon that can be added to the polymer matrix. The harder the polymer, the lower the user comfort as well as the more problems with polymer demolding and brittleness. Trial rubber formulations with higher carbon load and higher hardness resulted in more rejects in the molding process, since electrodes with very small pins or with long straight pins got easily stuck into the mold resulting in missing pins on the final electrode. Obviously, if one of the pin-cavities of the electrode mold is partially filled with stuck rubber also the subsequent moldings will have defect electrodes. Hence, this problem should be avoided. Rubber formulations with a higher carbon load and hardness resulted in 50% or more rejects especially for the more difficult designs (very small thin pins or long straight pins) while for the optimized electrode designs (conical pins, shorter pins) the fabrication yield was 100%.

**Figure 8.**
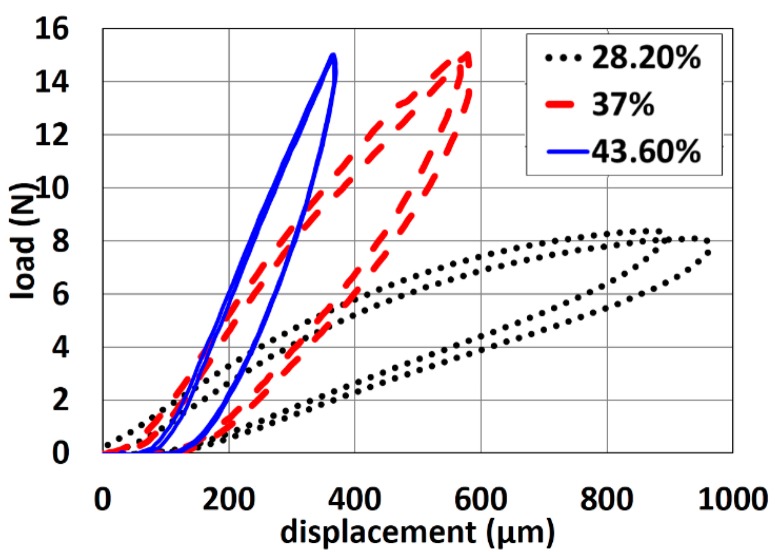
Load-displacement curves of pin-shaped electrodes containing various carbon content.

The load-displacement curves of pin-shaped electrodes containing various amount of carbon content are shown in Figure 8.

As shown, the slopes of loading and unloading curves increase with increasing carbon content. This reveals that the electrodes containing less carbon content are more flexible than the ones containing more carbon content. More results on the electrode flexibility as function of various shape designs can be found in previous work [27].

Elasticity is an intrinsic property of a material, while flexibility combines properties of the material and electrode shape. Overall, user comfort is reduced with a more rigid electrode. The flexibility of the electrodes makes them function like a spring. The pins of electrode bend to support the force from headset, elastic bands or other mounting devices. On the contrary, electrodes that are too flexible will not penetrate the hair to reach the scalp and could not support the weight and force of wearable devices. After analyzing these properties, electrodes with ∼50% carbon were used for ECG monitoring while electrodes with ∼45% carbon are used for EEG monitoring.

### 4.2. ECG Recordings

**Figure 9.**
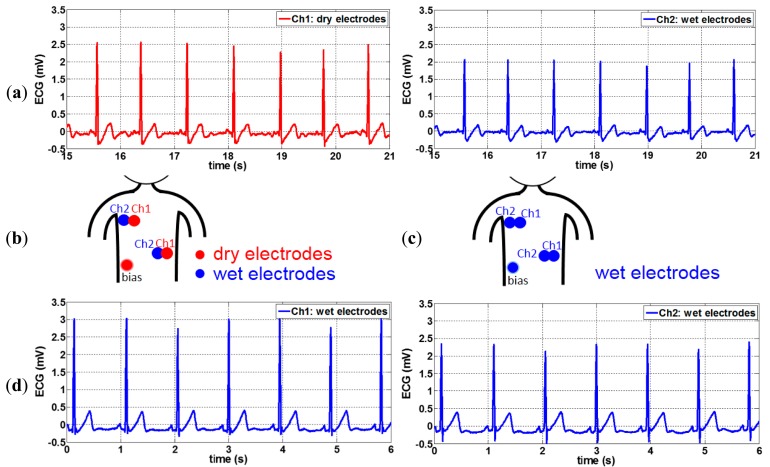
(**a**) Filtered ECG signals acquired by conductive polymer dry electrodes and by conventional wet electrodes; (**b**) Electrode locations using wet and dry electrodes; (**c**) Electrode locations using all wet electrodes; (**d**) Filtered ECG signals using all wet electrodes.

ECG measurements using polymer electrodes were performed as described in Section 3.2. It was found that polymer dry electrodes picked up more 50 Hz noise than conventional wet electrodes, however, these interferences could be easily filtered out. The filtered ECG signals acquired by conductive polymer dry electrodes and by conventional wet electrodes are shown in Figure 9a.

The shapes of both signals have high similarity and R peaks of both signals can be easily detected. T waves are also clearly seen. The correlation between both signals of Figure 9a is calculated, and a very high correlation (∼0.99) is obtained. This high correlation between wet and dry electrodes could be achieved when the subject does not move during the monitoring. Dry electrodes are more prone to motion artifacts due to less strong skin fixation comparing to wet electrodes and higher electrode/skin impedance. This can be improved by increasing the contact surface and applying better skin attachment approaches. The electrodes with 2 mm pins might be more suitable for subjects with some hair on the chest. For subjects without any hair on the chest, flat electrodes could be applied.

For this recording, the amplitude of the R peaks of the dry electrode signal is ∼0.6 mV higher than that of the wet ones due to the relative location of the electrodes. Even though the center of the wet and dry electrode is only 1.5 cm apart, as shown in Figure 3b, this difference in electrode location still affects the shape and amplitude of the ECG waves. This is easily to observe by an ECG recording using all wet electrodes placed on the same locations as the wet/dry electrodes of the previous test (see Figure 9b,c). The signals of the two wet channels were recorded simultaneously, as shown in Figure 9d. The amplitude of R peaks recorded by both wet channels differ by ∼0.5 mV, a variation only attributable to the small displacement between both sets of wet electrodes, and very close to the 0.6 mV variation seen for the dry/wet electrode pairs.

### 4.3. EEG Recordings

#### 4.3.1. EEG Recording by Using One Wet and One Dry Electrodes

**Figure 10.**
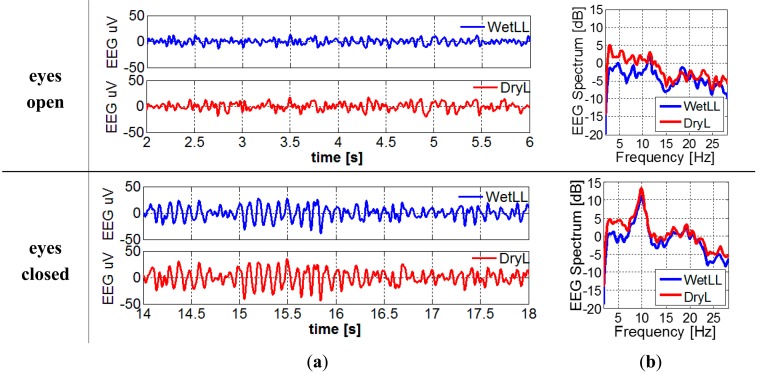
(**a**) Filtered EEG signals; (**b**) EEG spectrum of wet and dry electrodes when the eyes of subject were open and closed.

For the EEG recording with electrodes located in Figure 5b, filtered EEG signals and the EEG spectrum can be found in Figure 10a,b, respectively. The alpha waves can be clearly seen in the signals when the subject's eyes are closed. When converting the time domain EEG signal to frequency domain spectrum, the peaks at around 10 Hz become more obvious.

#### 4.3.2. Reference Recording by Using All Wet Electrodes

**Figure 11.**
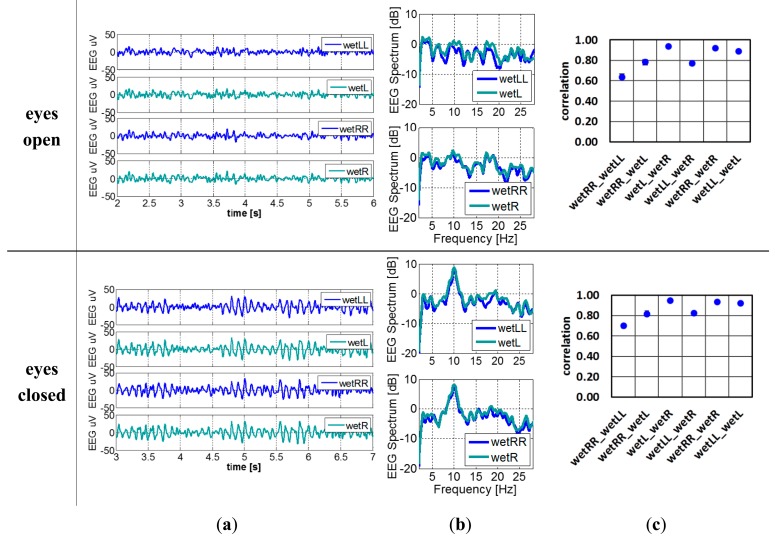
(**a**) Filtered EEG signals; (**b**) EEG spectrum; (**c**) Averaged correlation of reference recording when the eyes of subject were open and closed.

When replacing the dry electrode (dryL) to wet electrode (wetL) as shown in Figure 5c, the EEG signals and frequency spectrum and correlation between every pair of electrodes with the subject's eyes open and closed are shown in Figure 11. Alpha waves are easily detected as well. As expected, the electrodes positioned closer to each other have higher correlation. Hence since the wetLL-wetRR distance is the largest, their correlation the lowest. The ideal correlation with correlation coefficient 1 is unobtainable since it corresponds to two electrodes placed at exact the same location, hence covering a perfect identical brain region.

**Figure 12.**
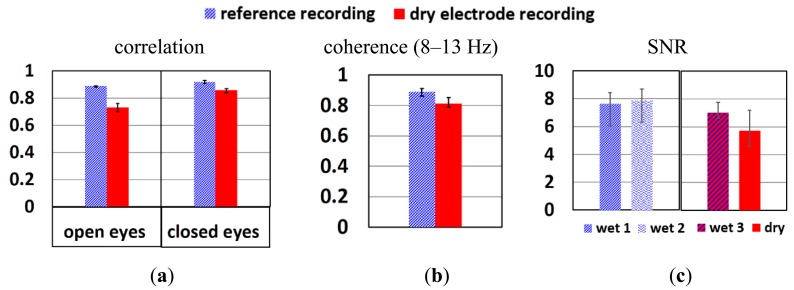
(**a**) Correlation when subject's eyes open and closed; (**b**) Coherence when subject's eyes closed; (**c**) SNR of each electrode of the wet gel electrodes (reference) and dry electrode recordings. The average value from three test segments of each recording condition is shown. The error bars indicate the best and worst result of the three segments.

To study if the signal quality of dry electrode reaches that of wet electrodes, the correlation, coherence and SNR between these two systems are compared. Figure 12a shows the correlation of wetLL-wetL (wet gel reference recording) and wetLL-dryL (from dry electrode recording) when the subject's eyes were open and closed. The wetLL-dryL signals show slightly weaker correlation than wetLL-wetL, but still above 0.7, which is the threshold of strong correlation. When there is a dominant signal in the segment of EEG recording, such as alpha waves, the correlation of wetLL-dryL improves and approaches that of wetLL-wetL. Figure 12b depicts the coherence of wetLL-weL in reference recording and wetLL-dryL in dry electrode recording when subject's eyes were closed. Due to the interest in alpha wave recording, only the coherence of signals from 8 to 13 Hz were studied. Signals of both pair of electrodes show coherence above 0.8. Figure 12c shows the SNR of wetLL, wetL of the wet gel reference recording (wet1, wet2) and wetLL, dryL (wet3, dry) of the dry electrode recording when subject's eyes were closed respectively. The signal of the dry electrode shows slightly lower SNR than that of wet electrodes.

As shown in Figure 10b, more low frequency signals (2–4 Hz) were picked up by dry electrodes. This might be the reason for theslightly lower correlation, coherence and SNR of dry electrodes compared to wet electrodes. However, the error bars show that in some recordings the signal quality of dry electrodes is comparable to that of wet ones. Longer stabilization time, better electrode fixation approach and cable shielding might further improve the signals.

### 4.4. User Comfort and Skin Irritation

In order to optimize for user comfort, the pins on the polymer electrodes were designed with rounded edges. Next, two dedicated experiments were performed to evaluate skin irritation using these electrodes: the first experiment evaluated short term use of the electrodes, while to second test focused on long tern usage of the electrodes.

#### 4.4.1. Short Term Usage of Polymer Electrodes

**Figure 13.**
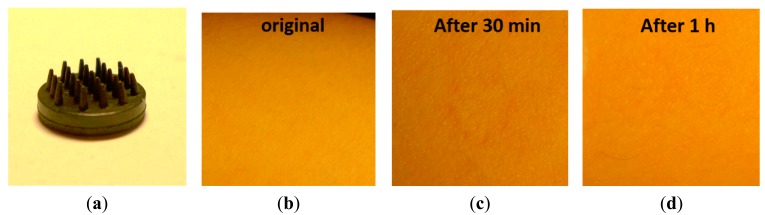
Skin irritation experiment for short term use of polymer electrodes (**a**) electrode with pins of 2 mm length; (**b**) Skin condition before and (**c**, **d**) after contact with short pin electrode.

**Figure 14.**
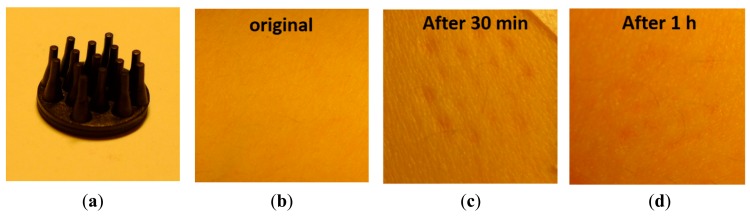
Skin irritation experiment for short term use of polymer electrodes (**a**) electrode with pins of 5 mm length; (**b**) Skin condition before and (**c**, **d**) after contact with long pin electrode.

Two electrode designs with pins of 2 mm and 5 mm length (see Figures 13a and 14a) were selected. These electrodes were placed on the skin with sufficient pressure to ensure low contact impedance, and the skin was evaluated after 30 min and 1 h of electrode contact. No pain nor discomfort was reported, and only a shallow impression on the skin was seen (see Figures 13c,d and 14c,d). During impedance evaluations and ECG monitoring, the electrodes were mounted using a sticker (see Figure 3a), while for EEG monitoring elastic bands are used (see Figure 5a). The pressure on the skin was at least sufficient for good electrode-skin contact, but often also higher, e.g. related to the size of the head and the elastic band for EEG monitoring. More than 10 subjects volunteered for the impedance, ECG and EEG evaluation, and hence the pressure of the electrodes on the skin varied from subject to subject. Most of the subjects did not feel important discomfort during and after the measurements. Shallow, painless pressure marks were often seen on locations with higher (hence not optimized) pressure caused by the elastic band being too tight, however, these marks disappeared fast. From these evaluations it is concluded that for maximum user comfort, it is important to mount the electrodes with a pressure not much higher than required for good contact.

#### 4.4.2. Long Term Usage of Polymer Electrodes

**Figure 15.**
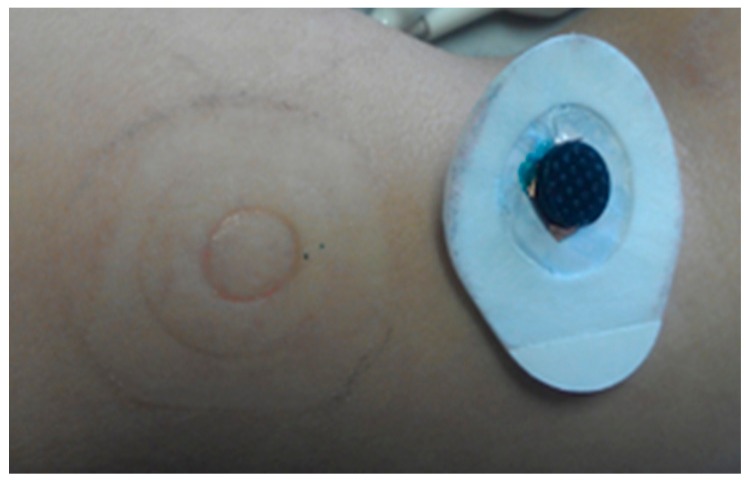
Skin condition after coin-shaped electrode/skin contact for 6 days.

A 6-day long-term test is performed using a coin-shaped electrode mounted on the upper arm skin using a non-breathable sticker. No major irritation or discomfort was reported for the total duration of the measurement, although some itching was observed, probably related to perspiration, since the non-breathable sticker precluded any ventilation. Further, some minor erythema (a painless and shallow pressure mark) was observed after 6 days of electrode contact, see Figure 15.

**Figure 16.**
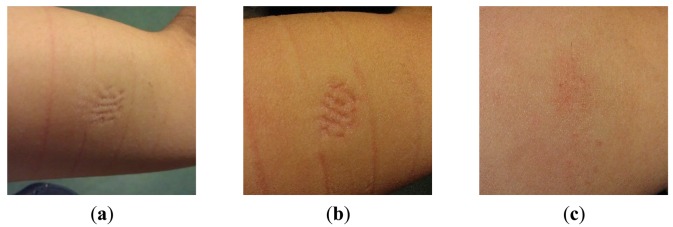
Skin condition (**a**) after 10 h of electrode/skin contact; (**b**) after 35 h of electrode/skin contact; (**c**) 4 h after the electrode was removed.

A second long-term measurement is performed using a pin-shaped electrode, contacting the skin for 60 h using an elastic band. No itching or pain was reported, although the subject was clearly observing the pressure of the electrode, described as very minor discomfort. Slight erythema was found after 10 h and ∼35 h, on the spots where the pins touched the skin, see Figure 16a,b. These marks are due to a rather high pressure from the bandage used for fixation. Even the elastic bandage showed pressure marks, as visible in Figure 16. The slight erythema found after 60 h was similar to that of after 35 h. After removing the electrode, the pressing mark faded fast, and was hardly visible anymore after 4 h, as shown in Figure 16c.

These results show that both coin-shaped and pin-shaped electrodes do not cause important irritation nor skin damage. Fixing the pin-shaped electrode by a tight elastic bandage is not ideal: the localized high pressure caused slight erythema. Obviously the elastic bands used in this work are for electrode evaluation purposes only. A dedicated EEG headset or ECG device which avoids high electrode pressure would be ideal for later clinical applications.

#### 4.4.3. Electrode Mounting Systems for EEG Recording

Elastic bands were used for EEG measurements in this work, since subjects reported that they were much comfortable than a conventional elastic EEG cap or commonly used silicone elastic wires. Compared to the generally used elastic cap, our elastic bands have a larger elasticity, and are also more comfortable since less skin area is covered, enabling perspiration to evaporate easily. Compared to the silicone elastic wires often used in hospitals in order to mount wet bridge electrodes (short-term EEG recording), our elastic bands distribute the pressure to larger skull area, causing less discomfort.

Eight subjects experienced these elastic bands in combination with the dry electrodes on their scalps for half an hour to several hours. They did not feel important discomfort during the tests. One subject had a large head, so the elastic bands were too short and caused important pressure. This person reported discomfort from the setup after using it for about 1 h.

As already mentioned before, the elastic bands used during this work are only selected for the purpose of evaluating the electrodes. Later, a dedicated EEG headset which avoids high electrode pressure should be combined with our polymer electrodes for future clinical applications.

## 5. Conclusions

Flexible polymer-based dry electrodes with high user comfort were fabricated. The polymer impedance decreases significantly with increasing carbon content. The skin-electrodes impedance for ∼50% carbon content is about 10-fold higher than that of conventional wet electrodes. Both the hardness and elastic modulus of polymer electrodes increase with higher carbon content. The polymer electrodes containing ∼45% of carbon were selected as the ones having the best compromise between electrical and mechanical properties. ECG signals acquired from these optimum electrodes are very similar to the ones obtained from wet electrodes. The EEG signals obtained by polymer electrodes show nearly the same correlation and coherence as wet electrode-signals, and only a slightly lower SNR is obtained. Alpha waves during “eyes closed” conditions can be observed clearly with dry electrodes. These results show that our polymer electrodes are promising alternatives for rigid dry electrodes and conventional gel electrodes.
